# Porn video shows, local brew, and transactional sex: HIV risk among youth in Kisumu, Kenya

**DOI:** 10.1186/1471-2458-11-635

**Published:** 2011-08-08

**Authors:** Carolyne Njue, Helene ACM Voeten, Pieter Remes

**Affiliations:** 1 Department of Public Health, Erasmus MC, University Medical Center Rotterdam, the Netherlands; 2 Social & Public Health Sciences Unit, Medical Research Council, Glasgow, UK; 3 Institute of Tropical Medicine, Antwerp, Belgium

## Abstract

**Background:**

Kisumu has shown a rising HIV prevalence over the past sentinel surveillance surveys, and most new infections are occurring among youth. We conducted a qualitative study to explore risk situations that can explain the high HIV prevalence among youth in Kisumu town, Kenya

**Methods:**

We conducted in-depth interviews with 150 adolescents aged 15 to 20, held 4 focus group discussions, and made 48 observations at places where youth spend their free time.

**Results:**

Porn video shows and local brew dens were identified as popular events where unprotected multipartner, concurrent, coerced and transactional sex occurs between adolescents. Video halls - rooms with a TV and VCR - often show pornography at night for a very small fee, and minors are allowed. Forced sex, gang rape and multiple concurrent relationships characterised the sexual encounters of youth, frequently facilitated by the abuse of alcohol, which is available for minors at low cost in local brew dens. For many sexually active girls, their vulnerability to STI/HIV infection is enhanced due to financial inequality, gender-related power difference and cultural norms. The desire for love and sexual pleasure also contributed to their multiple concurrent partnerships. A substantial number of girls and young women engaged in transactional sex, often with much older working partners. These partners had a stronger socio-economic position than young women, enabling them to use money/gifts as leverage for sex. Condom use was irregular during all types of sexual encounters.

**Conclusions:**

In Kisumu, local brew dens and porn video halls facilitate risky sexual encounters between youth. These places should be regulated and monitored by the government. Our study strongly points to female vulnerabilities and the role of men in perpetuating the local epidemic. Young men should be targeted in prevention activities, to change their attitudes related to power and control in relationships. Girls should be empowered how to negotiate safe sex, and their poverty should be addressed through income-generating activities.

## Background

Globally, the HIV epidemic is increasing faster amongst young women than young men and nowhere is this trend more apparent than in sub-Saharan Africa [1]. Statistics in sub-Saharan Africa remain disturbingly high, with 75% of all young people living with HIV being female [2-4]. The main form of HIV transmission is heterosexual sex. Of the young women aged 15-24, HIV prevalence is three times higher than HIV among their male counterparts. The contrasting HIV prevalence between boys and girls is a pattern observed in many parts of sub-Saharan Africa [4,5].

Kisumu town is found by the shores of Lake Victoria, is the capital of Nyanza province and the third largest town in Kenya. It is one of the most HIV/AIDS affected areas in Nyanza with a prevalence of 18.5% [6]. Nyanza province, in general, is the most severely affected, with HIV rates as high as 15%, which is double the national average [6,7]. Studies conducted in Kisumu in the late nineties show that HIV prevalence among girls was very high compared to boys (age group 15-19 years 23% versus 4%; age group 20-24 years 40% versus 13%) [6]. HIV prevalence among female sex workers was also very high, at 75% [8]. Although more recent figures for Kisumu are slightly lower, the numbers are still alarmingly high, and higher than in other parts of the country [9,6]. High HIV prevalence rates (30%) as well as very high rates of STIs have been noted among fishing communities along the shores of Lake Victoria [10,11]. These high rates of HIV/AIDS in Luo Nyanza have left 40% of children under 18 without one parent, and 11% without both parents [6,12]. Cultural norms such as wife inheritance and widow cleansing, polygamy, "jaboya" (in which female fishmongers develop sexual relationships with fishermen and middlemen in exchange for fish), and "chira" (a curse that comes from breaking certain taboos and traditions), continue to have a powerful hold on people in this lakeside province [10,13,14].

We conducted a qualitative study on the sexual behaviour of young people in Kisumu, to further explore results from an earlier population survey on factors determining the differential spread of HIV in four African cities: two with a relatively low and stable HIV prevalence (Cotonou and Yaoundé) and two where the spread of HIV has been explosive (Kisumu and Ndola) [15]. This study showed that girls in Kisumu had older sexual partners than boys and higher rates of herpes simplex type 2, which are both risk factors for HIV transmission. But most girls reported very few sexual encounters, and HIV prevalence was very high even among girls reporting one lifetime partner and few episodes of sexual intercourse. This may be due to underreporting, but also may indicate high transmission during loss of virginity [5]. Our aim was to deepen our understanding of the dynamics of sexual interactions of adolescents, in order to explain the high HIV prevalence among Kisumu youth in general and specifically among girls. We triangulated data from in-depth interviews, focus group discussions (FGD), and observations, to generate a holistic description of the contexts and dynamics of sexual interactions among youth.

## Methods

We conducted 150 in-depth interviews with adolescents aged 15-20 in Kisumu, held 4 FGDs, and performed 48 observations at places where youth spend their free time. The Institute of Tropical Medicine in Antwerp, Belgium, and the Ethical Review Board in Kisumu approved this study.

For the in-depth interviews, a convenience sample of 75 boys and 75 girls aged 15-20 years were interviewed at their households. Using the sampling framework of the multicentre study, quota sampling was used to ensure diversity in age, socio economic status (SES) of household, and education [9]. A qualitative interview guide was developed in English and translated into Swahili and Luo. Trained fieldworkers pre-tested the guide in communities neighboring the study sites. The interviews were held in Luo, Swahili or English by same-sex interviewers, and tape-recorded; they took about 45 minutes to one hour. Prior to the in-depth interviews, verbal informed consent/assent was obtained from all adolescents, in addition to parental consent for minors.

Four focus group discussions (FGDs) were held by same-sex interviewers in preparation for the in-depth interviews (i.e. with in-school males, in-school females, out-of-school males, out-of-school females). A topic guide was used, that was pre-tested for face and construct validity. The FGDs mainly focused on youth's attitudes, risk perception and socio-cultural norms regarding sexuality. Each FGD had 8 to 12 participants aged 15-20, and the discussions took about one and half hour.

We made 48 observations of young people's behaviour at places where youth spend their free time 'hanging around', such as nightclubs/bars, video halls, shopping malls, local brew dens, and funerals. We sought permission from the persons in charge and ensured confidentiality of all collected information. The field workers tried to get an inside view of reality without the participants' knowledge that they were being observed. Short notes were taken during the 2-to-3 hour observations when possible, and detailed notes were compiled afterwards describing the physical setting, the activities taking place, socio-demographics of participants (estimated age, gender), and their verbal and non verbal behaviour.

The audio-taped interviews and FGDs were transcribed verbatim and translated into English where necessary [16]. Data analysis of the in-depth interviews, FGDs and observations followed grounded theory principles, which allows analytical themes to emerge during the process of (re)reading transcripts and exploring and coding responses [17]. This approach is based on inductive analysis and consists of carefully reading/rereading interviews and observations, exploring and coding responses, and allowing new themes, issues and questions to emerge during the process. Using ATLAS.ti 4.1, a qualitative data analysis software program, the first and third author coded the transcripts, categorizing the data into themes, and identified the properties and dimensions of themes and subthemes. The following themes relating to risky sexual behaviour emerged from the interviews, FGDs and observations and are discussed below: young age at first sex, large age difference with male partners, multiple/concurrent partnerships, porn video shows, forced sex, low condom use, local brew/alcohol use and transactional sex. Where quotes are used in the Results section, they are from the in-depth interviews unless indicated otherwise.

## Results

Of the 150 interviewed adolescents, 55% were attending school (18 primary and 64 secondary school) whereas 45% were out-of-school youth (51 were unemployed and 17 were working in small micro-enterprise businesses, in domestic service, or as bicycle taxi operators). Over 85% of adolescents were born in Kisumu or had lived there for 10 years or more. The average age of adolescents interviewed in the study was 17.5 among boys and 17.0 among girls.

Of the youth interviewed, 79% of boys and 49% of girls reported they ever had had sex. Of these, 37% of boys (22/59) and 59% of girls (22/37) had their first sexual intercourse at age 15 or younger. A 15-year-old girl from a low-SES area stated: *"In this area of ours, many girls...get pregnant while they are very young because of starting sexual relationships very early, maybe like my age, you will find one has a boyfriend and later on she is made pregnant and then she is left which is not good."*

Most girls reported a large age difference with first and current partners. Sixteen of the 28 girls who mentioned the age of their first partner reported that he was 25 years or older. All girls with a current partner had a partner who was over 20 years old (ranging from 2 to 17 years older). One girl stated: *"...well if I am 20 I will go for a guy who is older than me by six years or something like three... because you cannot move *[date] *a guy who is the same age as you...." *Older men were preferred, as they proved to be more mature, could provide for their needs, and offer advice to solve problems. During the observations, older men were often seen with girls as young as 15.

Of the sexually active adolescents, over half of the girls reported having had 2-3 partners; over half of the boys indicated having had 3-5 partners and about a third reported more than 10 girlfriends. A few boys found it difficult to count all sexual partners: "*Some you can meet, you talk, have sex, then it just ends there so remembering them is difficult"*. Some of the bicycle taxi transporters reportedly had sex daily, with different partners. Girls mostly reported serial monogamy and rarely stated outright that they had concurrent partners. In contrast, many sexually active boys casually reported having overlapping partnerships: *"...the first is the one we talked about, ...the second is the one who was here, and the third is the one I connect with at the video hall." *Even among young men with a steady partner, it was common to have brief sexual encounters on the side, for example while at the video halls or attending a disco funeral. These 'disco funerals' are parties held by the relatives of a person recently deceased, in order to raise funds for the funeral. The disco funerals are characterised by loud music, singing, dancing, bidding games and risky sexual behaviour. Because we have described these events (in which Kisumu youth engage in risky sexual behaviour) in a separate paper, we do not elaborate on it here [18].

A third of the youth interviewed (49) report porn video shows either from personal attendance or as an influence on youth sexuality. Some also report watching porn at home or at the home of a friend: "*Whenever I used to go to the neighbour's to watch movies, she *[a 14-year old domestic worker] *could steal some tapes of 'pono', put them on*, *then try to convince me why don't we try that one*" (17 year-old boy). Video show halls are basically rooms with a television and VCR, they are popular leisure spots, where youth pay to watch movies. The fieldworkers did a dozen of random observations in video halls: 8 out of 12 were showing pornography at night. Most attendees were young men, but some girls also attended. The fee ranged from €0.05-0.15, and any youngster with a little money (even a 12-year old) was able to enter. The movies ranged from non-violent to violent pornography, and the scenes revolved around group sex, anal sex, and oral and vaginal intercourse. Youth said that the owner of a video hall most times disguises the announcement of a featured film and writes "on-por": in such a way youth know that "porno" will be shown. During one observation, several adolescents engaged in sex in the darkness of the hall. 

Reports of forced sex were many: 15 of 37 sexually active interviewed girls reported some degree of force/persuasion during first sex. Some girls were lured into secluded places such as a boy's cube [separate living quarter for boys], and were forced to have sex: "*This boy told me to visit him so when I went, he put the radio on... then later he just held me by force*." A 17-year-old boy reported: *"Say you've been dancing with her and you've told her that thing *[sex] *and she has refused, you just hold her and pull her by force till you go with her... to the bush or darkness where people don't go to..." *Another girl said that a boy grabbed and pinned her down and forced her to have sex at age 13. Some boys were said to waylay prostitutes and force them to have sex: *"Especially when it is night you meet a lot of girls hijacked by a group of men, being pulled to some place... they know these girls are prostitutes, they sell for money. But these groups of boys don't have any cash, they just get them and hijack them yah," *(FGD, out-of-school boys).

Few adolescents reported condom use at first sex (12 girls and 15 boys out of 96 sexually active adolescents). Condom use was neither common nor consistent: *"I have four girlfriends...I use a condom but not every time." *Reasons cited by boys for non-use included trust, discomfort, reduction of pleasure, and not having any condoms. For most girls, non-use was related to their limited ability to request condom use, ideals of intimacy and pleasure, and mixed messages about safety: *"Well... some don't use condoms because they tend to say that they will have side effects"*. Some young people also held the belief that very young boys and girls could not have STIs. Some adolescents only used condoms during the unsafe period in the menstrual cycle. Others reported they usually stop using condoms in a new relationship when they have become more used to each other or, as a few reported, after getting a negative HIV test.

Heavy alcohol intake and in particular illicit brews and drugs reinforced and mostly accompanied these sexual risk behaviours, as illustrated by this 15-year-old girl: *"So he asked me to have sex... but at first I didn't accept it ...so one day when I went, he bought this local brew 'kumi kumi' *[locally brewed gin] *and he persuaded me to take *[it], *afterwards I couldn't control myself and we found ourselves indulging in sex...." *Fieldworkers did random observations in local bars and brew dens and found that illicit brews such as chang'aa (cheap local spirit), kumi kumi and busaa (cheap local beer) and drugs such as khat, cannabis, and tobacco/betel quid were available especially in the low SES neighbourhoods. The illicit brew sold at a price of about €0.01 a cup. During interviews over a third (56) of youth interviewed (mostly boys) said they have gone to drink in these local brew dens. The brew was often made by widows who depend on the trade for their livelihood. It was reported that in environments where a parent(s) made local brews, girls started having sex at an early age and some progress to trade sex for money: "*... may be the mother is not steady, she moves from one man to another or may be she drinks...so when the woman is not around and men come to drink at her place, men start messing with her daughters... *(FGD, out-of-school girls). One girl said in an interview that she had her first sexual encounter at the age of fourteen, after her older sister described to her what the men were doing to her when she went to serve them (namely having sex for money). The younger girl got pregnant and gave birth at the age of fifteen. Another girl stated that she had sex several times with a client who promised to build her widowed mother a house.

Many interviewed youth reported to have had transactional sex. There were reports of young women going to local brew dens or disco funerals hoping the men would buy them drinks or give them money, in exchange of sexual favours. Often only a little money was required: buying a girl a soda was already seen as down payment for sex. Bicycle-taxi operators were reportedly popular, offering a few shillings, some food, or free transport to young women in exchange for sex: "*as long as you can buy her [something], maybe you buy her chips, you give her ten bob, you give her even a place to sleep, because she is not wanted at their place, you will just have sex with her*" (19-year old bicycle taxi-operator). Only occasionally did a boy receive gifts from his girlfriend(s). An 18-year-old girl from a low-SES area described how she decided to have sex with her 24-year old boyfriend: "...*after giving me gifts I just felt I should have sex with him." *Many of the young women interpreted receipt of money or gifts (like body lotion, soap, underpants and clothing) from their partners as loving gestures. Many young men acknowledged that their ability to provide for their girlfriends affected both the longevity and exclusivity of their relationships: "...*most girls go for men who can give everything, otherwise she'll get another person..." *(FGD, out-of-school boys). Eight of the 75 interviewed boys reported that that they had had contacts with a prostitute. Not much money was involved in such encounters: a 17 year-old young man from a middle-SES area said he paid €0.50 for two hours of sex with a prostitute when he was 16.

## Discussion

In this qualitative study on sexual behaviour of youth in Kisumu, Kenya, we found that the majority had sex at a young age, sometimes with multiple and concurrent partners, mostly without using a condom. Drugs and local alcohol often facilitated these encounters [19-22]. Some girls had transactional sex for material gain, not only for themselves but also for parents/guardians. Findings point strongly to the role of men in perpetuating the HIV epidemic (forcing sex, gang rape, multiple concurrent relationships). Many young people were exposed to pornography in video halls, which seemed to increase their sexual risk behavior. Peer influence was a great motivator for these risk behaviors.

There are a number of limitations that should be considered when interpreting the results. Because we used purposive quota sampling, it cannot be concluded that our results are fully representative for the total Kisumu youth population. Age was difficult to estimate from observation. We relied on participants' self-reports and there is a possibility of social desirability bias particularly among girls who may have underreported sexual experiences [23]. Caution is also suggested when generalizing our findings to other settings and populations, because the urban environment may have influenced youth norms regarding sexuality. Despite these limitations, the study was able to generate rich, descriptive data obtained through method triangulation, including new knowledge on a previously unstudied aspect of HIV risk- pornographic video shows. The high number of interviews held and the use of quota sampling ensured that the views of diverse youth (both girls and boys, in and out-of-school, in different SES) were incorporated.

Our findings point to gender-related power differences that expose young girls to HIV risk. Power-related differences manifest themselves not only in relationships, but also in the belief and structure of society [24]. For example, pre-marital and multi-partner sex, while typically portrayed (in Kisumu and elsewhere) as a breach of social norms, is also said to be a fundamental dimension of gendered social organization [25]. Men in settings like Kenya generally are expected to conform to a range of behavioural norms that confirm the hegemonic masculinity [26]. People consider it as a right and necessity, and part of the tradition, that men have more than one partner [27]. Pressure to be sexually adventurous and aggressive to prove manhood is quite pervasive in Africa. These norms allow men to have more sexual partners than women, encourage older men to have sexual relations with younger women, and increase the acceptance and justification of violence against women. It is not surprising therefore that our findings show that male partners force sex, perform gang rape, and have multiple concurrent relationships. Such norms and societal power relations consistently tend to disadvantage young women, as evidenced by the high incidence of transactional and coerced sex in many sub-Saharan countries [28].

The subordinate position of women, including the lack of control over finances and resources, has motivated girls to engage in multiple concurrent partnerships primarily for economic reasons, but at times the desire for love and sexual pleasure contributes to these partnerships [29-31]. Girls look at these partnerships in light of future plans, hoping for a steady relationship or marriage with an affluent older man. But also young men have a stronger socio-economic position than young women, enabling them to use money/gifts as leverage for sex. The material exchange accompanying sexual encounters may be interpreted as a loving gesture, but it may also express an unloving and calculating relationship.

The subordinate position of women may further force girls to endure abusive and violent relationships in order to secure economic gains. A high number of youth reported that either their mother or father had died, or sometimes both parents had passed away. These children are often left in the household with limited or no resources, where they often sink into poverty, forfeit their education, suffer from unattended psychological trauma, and become infected with HIV themselves [31,32]. A relationship with an older man (who is more likely to have a steady income than their age-mates) can provide them with the necessary livelihood support. There were reports of girls exchanging sex for money in order to feed their elderly parents and siblings, including access to material wealth such as expensive clothes and shoes.

These findings are consistent with recent studies in sub-Saharan Africa that show that factors such as cultural norms and gender roles place young women at risk of HIV infection [33,34]. They also show that men are expected to be dominant in a relationship, and many young girls may submit to men's sexual demands because they are expected to be subordinate, especially when they are (much) younger [33-35]. Moreover, such young girls are at a disadvantage in negotiating safe sex during such partnerships [36-38]. Young girls are coerced into sexual activities with older men for survival and to access material goods [34,39-42]. Sometimes the sexual exchange is to the benefit of the parent or guardian, and not the victim herself [43].

Our findings show how exposure to pornography in video halls encourages liberal sexual attitudes and behaviour among young people. The current phenomenon of porn video shows has not been described in literature. Studies on the effects of adults' exposure to pornography in developed countries show that repeated exposure to nonviolent pornography promotes more permissive sexual attitudes [44-46]. Findings also show that in the sprawling low SES neighbourhoods of urban Kisumu, alcohol use for young people is often synonymous with the locally brewed alcoholic beverages, due to their low price and wide availability. Alcohol is commonly used as a disinhibitor and a symbol of masculinity; it thus plays an important role in risky sexual behaviour [47]. Reports show the devastating effects of alcoholism: high rates of school dropouts, careless sexual behaviour, shattering of families, high rates of crime, and low productivity at work [47-49].

## Conclusions

Our findings have important implications for youth interventions. First, intervention strategies should engage young men in HIV prevention. Men in settings like Kenya generally control the terms and conditions of sexual relationships. Thus, we need better strategies to engage men, and effective interventions to change their attitudes and behaviours related to power and control in relationships. Interventions should promote more positive and safe actions like having respect for women, having one sexual partner, using condoms, and knowing their HIV status. Second, young girls should be empowered in several ways. They should be taught how to negotiate safe sex as part of the transactions in transactional sex. Their poverty should be addressed, e.g. through income generating activities or programs that keep them in school. For example, there are indications that programs which give loans to young women or youth groups, such as the Youth Enterprise Development Fund by the Kenyan government, are reducing the number of young women who have to exchange sex for money or who become teenage mothers [50]. Third, the government should regulate and monitor video shows and local brew dens, to prevent the exposure of young adolescents to pornography, drugs and alcohol. Legal action should be enforced on video hall and local brew den owners who promote pornography and/or alcohol consumption to youth. Instead, risk-free leisure activities should be developed for youth, such as sports facilities. Further research is needed to study the generalizability of our findings. More insight is needed on the effects of youth's exposure to pornography and the ways in which leisure spots such as video halls and local-brew dens can be used to promote safe sex practices.

## Competing interests

The authors declare that they have no competing interests.

## Authors' contributions

Each of the authors contributed to the article: *CN: *carried out the study, analysis and interpretation of data, drafting and revising of the manuscript; *HACM V*: revising the manuscript for substantial intellectual content and coordination, and *PR: *design, analysis and interpretation of data, involved in drafting and revising the manuscript. All authors have read and approved the final manuscript.

## Pre-publication history

The pre-publication history for this paper can be accessed here:

http://www.biomedcentral.com/1471-2458/11/635/prepub
